# Recurrent Laryngeal Edema Imitating Angioedema Caused by Dislocated Screw after Anterior Spine Surgery

**DOI:** 10.1155/2015/749463

**Published:** 2015-02-10

**Authors:** Piotr Wójtowicz, Tomasz Szafarowski, Ewa Migacz, Antoni Krzeski

**Affiliations:** ENT Department, Faculty of Medicine and Dentistry, Medical University of Warsaw, Stepinska Street 19/25, 00-739 Warsaw, Poland

## Abstract

The anterior cervical spine surgery is a common procedure to stabilize vertebrae damaged by various diseases. The plates and screws are usually used in the spine fixation. This kind of instrumentation may detach from the bones which is a rare but well-known complication. A 77-year-old male presented to the otorhinolaryngology department with throat pain, choking, and dysphagia. At first the angioedema was diagnosed and he was treated conservatively. The endoscopy revealed laryngeal edema, being more defined on the right side with right vocal fold paresis. CT scans showed the stabilizing plate with two screws attached tightly and the back-out of the third screw toward soft tissue of the neck. In the meantime, his condition deteriorated and he needed tracheotomy. In few days the surgical removal of the dislocated screw was performed successfully. Although two-month follow-up reported no obstruction of the larynx, the vocal folds paresis with gradual functional improvement was observed. Long-term complication of anterior spine surgery sometimes may suggest laryngeal angioedema at first. If the conservative treatment is ineffective and there is a history of anterior spine surgery, the clinicians should consider the displacement of the plate or screws in differential diagnosis.

## 1. Introduction

Anterior spine surgery is common and relieving procedure which is recommended for cervical disc herniation, trauma, neoplasm, spondylosis, osteomyelitis, and ossification of the posterior longitudinal ligament [1]. There are few techniques in use, including plates and screw fixation with or without bone implants [2]. As this surgery is generally based on stabilizing the spine with plate and screws, some complications regarding dislocation of the instrumentation may occur. The most common among them is the extrusion of the screw which results in pharyngoesophageal injury and perforation or prevertebral abscess [3]. The traction and friction between the retropharyngoesophageal wall and the cervical plate have also been reported. As the delayed pharyngoesophageal injury is a rare complication following anterior cervical spine surgery, the risk of perforation alone is up to 1.5% [2]. If dysphagia, odynophagia, and throat or neck pain are present just after surgery, the diagnosis is usually obvious. The same symptoms in the long term might be vague for clinicians [4].

We present the case of the delayed larynx injury and esophageal perforation, as a result of the screw dislodgement. The symptoms imitated laryngeal angioedema. The patient was treated successfully with a surgical removal of the screw. The esophageal perforation was managed conservatively.

## 2. Case

A 77-year-old Caucasian male presented to the otorhinolaryngology department with throat pain, choking, and dysphagia without dyspnea. He was unable to take any fluids. He visited other emergency departments three times before the admission, complaining about throat pain and dyspnea. Allergic angioedema was diagnosed; therefore the patient was treated with antihistamines with effect and partial remissions. His medical history included hypertension, nonsustained ventricular tachycardia, obstructive sleep apnea, gastroesophageal reflux disease, benign prostatic hyperplasia, left amblyopia, meningioma of left cavernous sinus, and anterior clinoid process. He underwent anterior spine surgery because of the cervical spondylosis 15 months before in different institution as well as septoplasty and turbinate surgery. He did not recall any allergic history.

**Figure 1:**
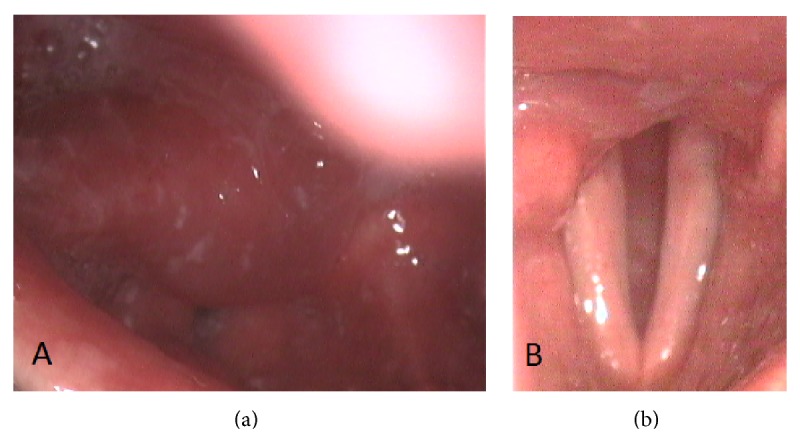
Fiberoscopy of the larynx preoperatively (a) and at four-week follow-up (b).

ENT examination indicated the angioedema of the larynx. Laboratory tests were normal except for leukocytosis (14,5 × 10^9^/L). He was admitted to the hospital and treated conservatively with ceftriaxone, dexamethasone, nonsteroidal anti-inflammatory drugs, and mucolytics. At the beginning, the condition of the patient improved, as he returned to oral fluids intake and dealt with the pain. On the seventh day of hospitalization he underwent laryngeal fiberoscopy. It showed the edema of aryepiglottic folds (Figure 1(a)), being more severe on the right side with vocal fold paresis. The neck CT was ordered due to diagnostic doubts.

**Figure 2:**
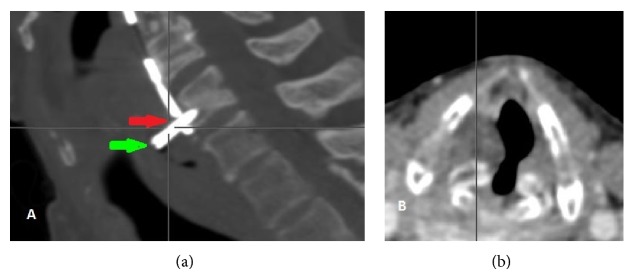
Sagittal reconstruction of the neck CT shows an extrusion of the screw at the C6 level (red arrow) and damaged postcricoid mucosa (green arrow) (a). 3D reconstruction of the plate and screws. 11 mm back-out of the one screw is noticeable (b).

CT scans showed the plate with two screws attached tightly to it at the C3-C5 vertebral level and 11 mm back-out of the third screw toward soft tissue of the neck at the C6 vertebral level (Figures 2(a) and 2(b)). The edema of surrounding tissue was observed. The presence of air was confirmed, so the esophageal perforation was suspected.

On the tenth day of the hospitalization, the rapidly increasing dyspnea was observed. The fiberoscopy revealed sustained edema of the aryepiglottic folds, right vocal fold paralysis, and left vocal fold paresis. The obstruction of the larynx precluded the intubation; therefore the tracheotomy with local anesthesia was performed. The patient was supplied with nasogastric tube.

**Figure 3:**
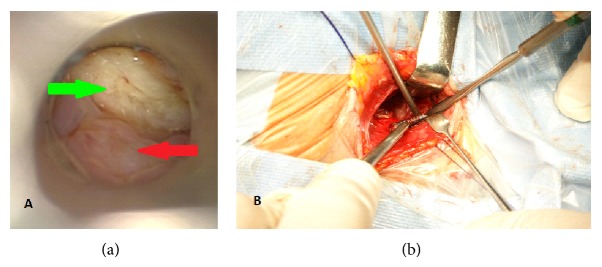
Endoscopic view of the necrosis of mucosa on the back of lamina of cricoid cartilage (green arrow) and esophageal mucosa (red arrow) (a). Intraoperative photograph shows the screw during removal (b).

Next direct laryngoscopy done four days later showed necrosis of posterior part of cricoid cartilage and the granulation tissue in the clinging esophagus (Figure 3(a)). No evidence of foreign body (screw) was confirmed. The specimens were obtained for pathological analysis which revealed focal purulent inflammatory process.

The decision about the neck revision under general anesthesia was taken. Skin incision and dissection of the postoperative scar and inflammatory tissue were performed. After localization of the plate, the loose screw was removed (Figure 3(b)). No esophageal fistula was found. JP drain was placed and all tissues were closed. 20 days following the surgery the nasogastric tube was taken out and the patient used a plug to close the tracheostomy temporarily.

The two-month follow-up reported no symptoms of the laryngeal obstruction. The endoscopy of the larynx revealed vocal fold paresis with gradual functional improvement (Figure 1(b)). The patient was scheduled for surgical closure of the tracheostomy.

## 3. Discussion

Anterior cervical spine surgery with a plate fixation is a well-established procedure intended for conditions such as trauma, tumors, or spondylotic myelopathy with 98% effectiveness to achieve a good fusion [2].

Most complications regarding the extrusion of the screw are localized in the esophagus. They range from asymptomatic perforation to acute rupture of esophagus [5]. There are also reported cases of migrating screw successively eliminated through the gastrointestinal tract. Only few cases of obstruction and damage of the airways are described [1].

If there is a suspicion of pharyngoesophageal perforation or larynx injury, it is crucial to establish the diagnosis as soon as possible. The relevant treatment introduced within 24 hours significantly increases the chances of full recovery.

Presented case was a long-term complication of the anterior cervical spine surgery. At the beginning ENT examination tests suggested the angioedema of the larynx; therefore the patient was treated intravenously with antibiotics and steroids. The laryngeal endoscopy showed the edema of aryepiglottic folds, being more severe on the right side with vocal fold paresis, so CT of the neck was done. In the meantime, the condition of the patient deteriorated and the urgent tracheostomy was necessary. As CT scans showed the back-out of the screw, the direct laryngoscopy was performed, but there were no signs of the foreign body localized submucosally. When the condition of the patient was finally stable, the removal of the screw was performed with neck incision.

Although dislocation of the screw is well known in literature, it is a rare complication [6]. Therefore the diagnostic process may be delayed and the condition of the patient may deteriorate because of the inaccurate treatment. There are risk factors which predispose the patients to the dislodgement of instrumentation (cervical neck trauma and esophageal diverticula) [7], but it is usually a spontaneous process. In some cases the removal of the screw is not sufficient because the damage of the tissue is very extensive. It requires additional complex procedures such as reinforcement of closure of perforation with a vascularized flap in order to facilitate healing [4].

## 4. Conclusion

Long-term complication of anterior spine surgery may suggest laryngeal angioedema, as described in this case. If the conservative treatment is ineffective and there is a history of anterior spine surgery, the clinicians should include displacement of the plate or screws in differential diagnosis.

## References

[1] Duransoy Y. K., Mete M., Zengel B., Selçukı M. (2013). Missing screw as a rare complication of anterior cervical instrumentation. *Case Reports in Orthopedics*.

[2] Solerio D., Ruffini E., Gargiulo G. (2008). Successful surgical management of a delayed pharyngo-esophageal perforation after anterior cervical spine plating. *European Spine Journal*.

[3] Martínez-Lage J. F., Felipe-Murcia M., Azorín L. M.-L. (2007). Late prevertebral abscess following anterior cervical plating: the missing screw. *Neurocirugia*.

[4] Phommachanh V., Patil Y. J., McCaffrey T. V., Vale F., Freeman T. B., Padhya T. A. (2010). Otolaryngologic management of delayed pharyngoesophageal perforation following anterior cervical spine surgery. *Laryngoscope*.

[5] Mueller O., Haag S., Sure U., Naglatzki R. (2011). A loose screw or two. *The Lancet*.

[6] Gaudinez R. F., English G. M., Gebhard J. S., Brugman J. L., Donaldson D. H., Brown C. W. (2000). Esophageal perforations after anterior cervical surgery. *Journal of Spinal Disorders*.

[7] Patel N. P., Wolcott W. P., Johnson J. P. (2008). Esophageal injury associated with anterior cervical spine surgery. *Surgical Neurology*.

